# Mitochondrial Bioenergetics of Extramammary Tissues in Lactating Dairy Cattle

**DOI:** 10.3390/ani11092647

**Published:** 2021-09-09

**Authors:** Victoria Favorit, Wendy R. Hood, Andreas N. Kavazis, Patricia Villamediana, Kang Nian Yap, Hailey A. Parry, Amy L. Skibiel

**Affiliations:** 1 Department of Animal, Veterinary and Food Sciences, University of Idaho, Moscow, ID 83844, USA; favo4940@vandals.uidaho.edu (V.F.); pvillamediana@uidaho.edu (P.V.); 2 Department of Biological Sciences, Auburn University, Auburn, AL 36849, USA; wrhood@auburn.edu (W.R.H.); kny0004@auburn.edu (K.N.Y.); 3 School of Kinesiology, Auburn University, Auburn, AL 36849, USA; ank0012@auburn.edu (A.N.K.); hap0017@auburn.edu (H.A.P.)

**Keywords:** oxidative phosphorylation, cellular respiration, metabolism, metabolic adaptation, liver, skeletal muscle

## Abstract

**Simple Summary:**

The nutrient and energy requirements of lactation are among the greatest required by any physiological process in the female mammal. The mammary gland and extramammary tissues undergo metabolic adaptations that coordinate changes in energy availability and nutrient partitioning that enable milk synthesis. Mitochondria are largely responsible for energy production in cells and their importance in milk synthesis has long been appreciated. However, mitochondrial adaptations across lactation are understudied, particularly for extramammary tissues with supporting roles in milk synthesis. Tracking mitochondrial function in dairy cattle across lactation, we found that the efficiency of energy production in the liver was elevated in the presence of fat-based substrates as the milk yield was increasing. In skeletal muscle, mitochondrial function showed little change across lactation and was not associated with milk production, suggesting that energy efficiency in this tissue is consistent regardless of the metabolic demands of lactation. A better understanding of mitochondrial bioenergetics during lactation may provide insight into the etiology of metabolic diseases during the transition period and low milk supply.

**Abstract:**

Lactation is physiologically demanding, requiring increased nutrient and energy use. Mammary and extramammary tissues undergo metabolic changes for lactation. Although it has long been recognized that mitochondria play a critical role in lactation, the mitochondrial adaptations for milk synthesis in supporting tissues, such as liver and skeletal muscle are relatively understudied. In this study, we assessed the mitochondrial function in these tissues across lactation in dairy cattle. Tissue biopsies were taken at 8 ± 2 d (early, *n* = 11), 75 ± 4 d (peak, *n* = 11) and 199 ± 6 d (late, *n* = 11) in milk. Early lactation biopsies were harvested from one group of cows and the peak and late biopsies from a second cohort. Milk yield (MY) was recorded at each milking and milk samples were collected for composition analysis. Mitochondrial efficiency was quantified as the respiratory control ratio (RCR), comparing maximal to resting respiration rates. Liver complex II RCR was positively associated with MY. Liver ROS emission increased across lactation whereas liver antioxidant activity was similar across lactation. No change was detected in skeletal muscle RCR or ROS emission, but muscle GPx activity decreased across lactation and muscle SOD was negatively associated with MY. Muscle oxidative damage was elevated at early and late lactation. Across lactation, genes involved in mitochondrial biogenesis were upregulated in the liver. Our results indicate that during lactation, liver mitochondrial biogenesis and efficiency are increased, which is associated with greater milk yield. In contrast, the mitochondrial efficiency in skeletal muscle remains consistent across lactation, but undergoes oxidative damage, which is associated with reduced antioxidant activity.

## 1. Introduction

Lactation is a highly energetically demanding process that requires metabolic adaptations in the mammary gland and extramammary tissues [1]. The energy requirements for lactation are even greater than those for gestation, increasing to peak lactation before declining towards the dry off period [1,2,3]. At the onset of lactation, dairy cattle enter a state of negative energy balance (NEB) where feed intake and nutrient availability are outpaced by the requirements of lactation and maintenance [4]. This causes several physiological changes, such as mobilization of adipose tissue [5], increased hepatic glycogen mobilization and gluconeogenesis [1,6], and increased skeletal muscle proteolysis and reduced protein synthesis [7,8]. These metabolic adjustments allow tissues to shuttle nutrients to the mammary gland for milk synthesis [6,9]. Insufficient metabolic adjustment leads to health issues and metabolic disease that may disrupt milk production across the whole lactation [10].

Adaptations in mammary mitochondrial physiology are integral to support the lactating mammary gland and thus are linked to milk production and cow health [11,12]. Mitochondria are biosynthetic, fueling the production of precursors, such as amino acids and fatty acids, for milk biosynthesis [13]. As such, maternally inherited polymorphisms in mitochondrial DNA (mtDNA) are associated with the energy density and fat composition of milk in dairy cattle [14]. Mitochondria also produce up to 90% of ATP available for use by tissues [15]. The mammary mitochondrial density and mtDNA copy number increase, thus allowing for greater ATP production in early lactation as mammary energy demand and milk output are rising [15,16]. In late lactation, the mammary mitochondrial density, copy number, protein expression of electron transport system (ETS) subunits and ATP synthesis decrease [3,15,17].

Apart from a study in bovine liver showing a higher number of mitochondria in late lactation relative to early lactation [3], little is known about mitochondrial adaptations in extramammary tissues, such as liver and skeletal muscle, across lactation. However, studies highlighting differences between lactating and non-lactating animals and recent molecular work provide some insight into the mitochondrial bioenergetics of these tissues during lactation. For example, the efficiency of mitochondrial respiration is higher in liver and skeletal muscle of lactating rodents, relative to nonreproductive and nonlactating groups, [18,19]. The genes and transcription factors involved in mitochondrial regulation in extramammary tissues of dairy cattle, are upregulated during the transition period to facilitate changes in nutrient metabolism and increase bioenergetic capacity [20,21].

The majority of mitochondrial proteins are nuclear-encoded, relying on coordinated signaling pathways between the mitochondria and nucleus [22]. Mitochondria communicate with the nucleus through signaling pathways involving reactive oxygen species (ROS) and ATP that alter transcription of nuclear encoded mitochondrial proteins [23]. However, excess ROS production can overwhelm antioxidant defense systems and result in oxidative stress and tissue damage [24]. It was proposed that elevated energy demands during lactation induces greater oxidative stress due to higher rates of oxidative phosphorylation [25]. However, the outcomes of studies in dairy cattle are equivocal, with some supporting and others rejecting this hypothesis [26,27].

In this study we assessed temporal changes in mitochondrial biogenesis and function and mito-nuclear regulation in skeletal muscle and liver in relation to milk synthesis across lactation. We hypothesized that changes in mitochondrial activity would mirror temporal variation in milk yield (MY), and consequently energy demand. Further, we predicted that enhanced oxidative capacity of mitochondria, particularly in early and mid-lactation, would result in greater oxidant emission, culminating in oxidative damage.

## 2. Materials and Methods

### 2.1. Study Animals and Tissue Biopsies

This study involved lactating multiparous Holsteins at the University of Idaho Dairy Center that calved in the fall. All animal procedures were approved by the Institutional Animal Care and Use Committee (IACUC) at the University of Idaho. Cows were fed a total mixed ration (TMR) for lactating cattle and milked four times daily for the first 42 d in milk (DIM), then twice daily for the remainder of lactation. Skeletal muscle and liver biopsies were collected at early (8 ± 2 d), peak (75 ± 4 d) and late (199 ± 6 d) lactation. Samples obtained at peak and late lactation were from the same 11 cows whereas the early timepoint samples were collected from an additional set of 11 cows, due to sampling and breeding constraints. Cows from both cohorts were similar in mature equivalent milk yield (12,177 ± 1565 kg vs. 12,027 ± 758 kg) and parity (3.3 ± 0.47 vs. 2.5 ± 0.21). Cows at all timepoints were similar in body weight (early 643 ± 20 kg, peak 631 ± 23 kg, late 646 ± 23 kg) and body condition score (early 3.30 ± 0.08, peak 3.25 ± 0.15, late 3.30 ± 0.13 on a 5.0 scale). A sample size of 10 cows per timepoint was determined by power analysis (85% power, alpha level of 0.05) and based on previous studies of mitochondrial function in animal models [15,18,19]. All cows were healthy and showed no signs of mastitis or other ailments. For biopsy, cows were lightly anesthetized with an intravenous injection of xylazine HCL (100 mg/mL concentration; dose, 0.025 mg/kg). The biopsy fields were shaved and cleaned with iodine and isopropanol. Local anesthesia was achieved by subcutaneous and intramuscular injections of lidocaine around the biopsy site. Liver samples were collected using an 8 mm stainless steel liver puncture biopsy tool on the animal’s right side through the 10th or 11th intercostal space on an approximate line from the hooks to the elbow (*n* = 32, one liver sample was not obtained). Skeletal muscle was collected by blunt dissection of the biceps femoris muscle approximately 10–20 mm deep and alternating left and right legs with each biopsy (*n* = 33).

### 2.2. Tissue Processing

Tissues were aliquoted into cryovials, and flash frozen in liquid nitrogen or placed in RNAlater and stored at −80 °C for later analyses. An additional 1.0 g of liver was minced on ice in 10 mL of liver isolation buffer (250 mM sucrose, 5 mM HEPES and 1 mM EGTA) and 1.0 g of skeletal muscle was minced in 10 mL of muscle isolation buffer with BSA (100 mM KCl, 40 mM Tris-HCl, 10 mM Tris Base, 1 mM MgCl_2_, 1 Mm EGTA, 0.2 mM ATP and 0.15% BSA).

### 2.3. Mitochondrial Isolation

Mitochondria were isolated from minced tissue as detailed in [28,29,30]. Liver was homogenized with 5–6 passes with a Potter-Elvehjem PTFE tissue grinder (DWK Life Sciences, Millville, NJ, USA) and centrifuged in 50 mL round-bottom centrifuge tubes for 10 min at 500× *g* at 4 °C. The supernatant was filtered through gauze and centrifuged for 10 min at 3500× *g* at 4 °C. The resulting pellet was resuspended in 10 mL of liver isolation buffer before centrifuging a final time for 10 min at 3500× *g* at 4 °C. The final pellet was resuspended in 0.25–0.35 mL of mannitol sucrose (220 mM mannitol, 70 mM sucrose, 10 mM Tris-HCl and 1 mM EGTA). Isolated mitochondria were homogenized by 5–10 passes with a Dounce homogenizer (Corning, Glendale, AZ, USA).

Skeletal muscle tissue was homogenized using a Polytron Biotrona homogenizer (Kinematica, New York, NY, USA). Then, 5 mg trypsin/g of wet tissue was added and after a 7 min incubation on ice, 10 mL of muscle isolation buffer with BSA was added to stop digestion. After centrifuging for 10 min at 500× *g* at 4 °C, the supernatant was filtered through gauze and centrifuged for 10 min at 3500× *g* at 4 °C. The pellet was resuspended in 10 mL of muscle isolation buffer with BSA and the suspension centrifuged again for 10 min at 3500× *g* at 4 °C. The pellet was resuspended with muscle isolation buffer without BSA (100 mM KCl, 40 mM Tris-HCl, 10 mM Tris Base, 1 mM MgCl2, 1 mM EGTA, 0.2 mM ATP) and centrifuged again for 10 min at 3500× *g* at 4 °C. The final pellet was suspended in 0.2–0.25 mL of mannitol sucrose. The isolated mitochondria were homogenized using 5–10 passes with a Dounce homogenizer.

### 2.4. Mitochondrial Respiration

Respiration was performed on freshly isolated mitochondria in Oxytherm+ respiration chambers (Hansatech Instruments, Ltd., Pentney, UK) using substrates of complex I and II of the ETS as previously described [30,31,32]. Respiration buffer (100 mM KCl, 50 mM MOPS, 10 mM KH2PO4, 20 mM glucose, 10 mM MgCl2, 1 mM EGTA and 0.20% BSA) was added to each chamber (925 μL for complex I and 943 μL for complex II) followed by 40 μL of isolated mitochondria. For complex I, 10 μL of 2 mM malate, 10 mM glutamate, and 2 mM pyruvate were added followed by 5 μL of ADP. For complex II, 2 μL of rotenone (4 mg/mL in DMSO) and 10 μL of 5 mM succinate was added followed by 5 μL of ADP. The respiratory control ratio (RCR) was calculated as the state 3 to state 4 slopes. State 3 occurs immediately after the addition of ADP and state 4 is the following plateau as oxygen utilization slows to the resting respiration rate and ADP is phosphorylated to ATP. State 3 and state 4 values were normalized to mitochondrial protein content, which was quantified using a BCA Protein Assay (Thermo Fisher Pierce, Waltham, MA, USA, #23225). RCR could not be computed for 4 liver complex I samples (1 early, 2 peak, 1 late), 2 liver complex II samples (both early), 8 muscle complex I samples (2 early, 3 peak, 3 late), and 7 muscle complex II samples (2 early, 2 mid, 3 late) due to non-viable mitochondria.

### 2.5. Milk and Blood Collection

Milk yield data for the day before each biopsy were extracted from the DairyPlan database at the University of Idaho Dairy Center. Composite milk samples from all milkings were taken the day before biopsy for composition analysis of fat, protein, and lactose via infrared technology (Northwest Labs, Jerome, ID, USA). Blood was collected from the coccygeal vein prior to each biopsy into serum and sodium heparin vacutainer tubes. Serum and plasma were separated from whole blood and stored at −20 °C until blood metabolite analysis.

### 2.6. Blood Metabolites

Plasma glucose concentrations were quantified using Autokit Glucose (Colorimetric) (Wako Fujifilm, Lexington, MA, USA, #99703001) according to the manufacturer’s instructions. Plasma BHB concentrations were quantified using Autokit 3-HB (Colorimetric) (Wako Fujifilm, #417-73501, 413-73601, 412-73791) following the manufacturer’s instructions. Plasma for β-hydroxybutyrate (BHB) was diluted 1:10 with 0.9% saline. Serum nonesterified fatty acid (NEFA) concentrations were quantified according to the manufacturer’s protocol using the NEFA kit (Colorimetric) (Wako Fujifilm, #999-34691, 995- 34791, 991-34891, 993-35191, 276-76491). All samples were run in duplicate. The inter-assay CV was less than 15% for all metabolite assays. The average intra-assay CV was 5.9%.

### 2.7. Mitochondrial Oxidant Emission

ROS emission was quantified using the Amplex™ Red Hydrogen Peroxide/Peroxidase Kit (Invitrogen™, Carlsbad, CA, USA, #A22188) with slight modification to the manufacturer’s protocol. Liver mitochondria were diluted 1:4 in mannitol sucrose while skeletal muscle mitochondria were run undiluted. A quantity of 10 μL of each mitochondrial suspension was assayed in 190 μL of succinate buffer containing 500 mM KCl and 50 mM MgCl_2_ to measure peroxide production.

### 2.8. Tissue Antioxidant Activity

Antioxidant activity was measured for liver and skeletal muscle using the Superoxide Dismutase Activity Assay Kit (Colorimetric) (Abcam, Cambridge, MA, USA, #ab65354), which quantifies activity of all SOD isoforms, and Glutathione Peroxidase Assay Kit (Colorimetric) (Abcam, Cambridge, MA, USA, #ab102530) according to the manufacturer’s instructions. Samples for SOD activity were prepared using 10 mg of tissue rinsed in ice cold PBS, homogenized using a Dounce homogenizer in 250 μL of buffer (0.1 M Tris HCl, pH 7.4; 0.5% Triton X-100, 5 mM β-ME, 0.1 mg/mL PMSF) and centrifuged for 5 min at 14,000× *g* at 4 °C to collect the supernatant. Tissue samples for the GPx assay were prepared using 100 mg of tissue rinsed in ice cold PBS and homogenized in 200 μL of kit assay buffer with a polytron and centrifuged for 15 min at 10,000× *g* at 4 °C to collect the supernatant. The supernatant was used in both assays to quantify antioxidant activity with all samples run in duplicate. The average inter-assay CV was 10.5% and the average intra-assay CV was 2.2%.

### 2.9. Oxidative Damage

Oxidative damage to liver and skeletal muscle was quantified using the Oxiselect™ Protein Carbonyl ELISA Kit (Colorimetric) (Cell Biolabs, INC., San Diego, CA, USA, #STA-310) and the Colorimetric Lipid Peroxidation (MDA) Assay (Abcam, #ab118970). For protein carbonyl, 60 mg of tissue was homogenized with a polytron in 1 mL of buffer (990 μL PBS and 10 μL PMSF) and centrifuged for 10 min at 10,000× *g* at 4 °C to collect the supernatant. The supernatant was analyzed for protein content using a BCA assay (Thermo Fisher Pierce™ BCA Protein Assay, #23225) and diluted to a concentration of 10 μg/mL using PBS to normalize protein content across samples. The ELISA was performed according to the manufacturer’s protocol with a final incubation time of 15–20 min before adding the stop solution. For MDA, 20 mg of tissue was rinsed in ice cold PBS and homogenized using a polytron in 300 μL ddH2O with 6 μL BHT. After homogenization, 300 μL of 2 N perchloric acid was added and then centrifuged (10 min, 13,000× *g*, 4 °C) to precipitate the protein The assay was performed according to manufacturer’s protocol with undiluted samples run in duplicate. The average inter-assay CV was 9.4% and the average intra-assay CV was 13.1%.

### 2.10. Adenine Nucleotides

Tissue adenosine nucleotide concentrations were quantified with an ATP/ADP/AMP Assay Kit (Biomedical Research Service Center University at Buffalo, State University of New York, Buffalo, NY, USA, #A-125). A quantity of 50 mg of tissue was homogenized in 200 μL of 10% TCA and the samples were deproteinated following the manufacturer’s protocol. The samples were assayed in duplicate using a sample volume of 2.5 μL.

### 2.11. RNA Extraction and cDNA Synthesis

Approximately 50 mg of liver tissue was homogenized in 1 mL TRIzol and RNA extracted using the RNeasy Plus Universal Mini Kit (Qiagen, Germantown, MD, USA #73404). Approximately 70 mg of skeletal muscle tissue was homogenized and RNA extracted using the TRIzol™ Reagent (Invitrogen ThermoFisher Scientific, Waltham, MA, USA #15596026) according to the manufacturer’s protocol. RNA quality and concentration were measured using a Nanodrop spectrophotometer (ND-1000; Thermo Scientific). cDNA was synthesized from 1 μg of RNA using iScript Reverse Transcription Supermix for qRT-PCR (BioRad, Berkeley, CA, USA #1708840) with a Veriti 96-well Thermal Cycler (Applied Biosystems, Watham, MA, USA) and the following run protocol, 25 °C for 5 min, 46 °C for 20 min, 95 °C for 1 min. Liver cDNA was diluted 1:4 in ddH2O. Skeletal muscle cDNA was not diluted.

### 2.12. qRT-PCR

Primer sequences were extracted from published literature or were developed in NCBI Primer BLAST (Table 1). Reaction mixes consisted of 6.25 μL PowerUp™ SYBER™ Green Master Mix (Applied Biosystems, #A25742), 500 nM each of forward and reverse primers, 2.5 μL of cDNA, and ddH2O to a final volume of 10 μL. Primers were validated for linearity and specificity by conducting 5-fold serial dilutions and through melt curve analysis, respectively. qRT-PCR was performed using a ViiA 7 RT-PCR system (Applied Biosystems) with the following protocol: 50 °C for 2 min, 95 °C for 2 min, forty cycles of 95 °C for 2 s and 60 °C for 30 s, and a melting curve of 95 °C for 15 s, 60 °C for 1 min, and 95 °C for 5 s. Efficiency ranged from 90 to 113 (avg. 103 ± 6.83) and R2 from 0.92 to 1.0 (avg. 0.98 ± 0.024). A negative (no-template) and a positive control were included in each plate and all samples were run in duplicate. Bovine GAPDH, RPS9, and RPS18 were used as reference genes.

**Table 1:** Primer information for qRT-PCR genes.

Accession No.	Gene	Sequence (5′-3′)	PCR Product Length
NM_177945	PGC-1α *	F: GTACCAGCACGAAAGGCTCAA R: ATCACACGGCGCTCTTCAA	120
NM_001034036.1	PPARα	F: GTGAGGGCTGCAAGGGTTTC R: TCTCAGATCTTGGCATTCGTCCA	186
NM_001083636.1	PPAR*d*	F: TGCACATGGTACTCACGCAG R: TGCTCCATGGCTGACTTCCC	172
NM_001109802.2	AMPKα	F: AACCTGAAAACGTCCTGCTTGA R: TGGACCAGCATACAATCTTCCTG	158
NM_001024558.1	AMPKβ	F: ACGACCCTTCCGAGCCAGTA R: CGGTGGGGAACTGGACAACT	149
NM_174586.2	AMPKγ	F: TGGTGGATGAGAAAGGGCGTG R: ATCGATGTTGCAGGGCTTTGG	120
NM_001098002.2	NRF1	F: CTGATGGCACTGTCTCGCTT R: GCCCAGTTTTGTTCCACCTCT	145
NM_001075437.2	NRF2α	F: GGACGGCTGTAGGTGAGACA R: GTGCACTCTGCTTTCTCTGTTCC	142
NM_001034016.2	TFAM	F: GTTTTCAGGAAGCTAGGGATGGC R: ACTTCCATCATTTGTTCCTCCCAAG	169
NM_001114610.1	TFB1M	F: TCAAAAGGAAGTGGCAGAGAGACT R: TGAAGGGCTGCTTGATCCTGG	200
NM_001038127.2	TFB2M	F: TTGGGAGTGAAAGCACATCCT R: TTGCCGTTAGTTTCCGGCAT	193
NM_001206669.1	SIRT3	F: AACATCGACGGGCTCGAGAG R: CATCACGTCGGCCCAGAAGT	135
NM_201527.2	SOD2	F: GGGTTGGCTCGGCTTCAATA R: TGCTCCCACACGTCAATCCC	120
NM_174076.3	GPx1	F: ACACCCAGATGAATGACCTGCA R: TCGCCATTCACCTCGCACTTT	193
NM_001035386.2	CAT	F: TTCGCTTCTCCACTGTTGCTGG R: CAGGTGCGTTTGAGGGTTTCT	197
NM_001101152	RPS9	F: GGAGACCCTTCGAGAAGTCC R: CTTTCTCATCCAGCGTCAGC	84
NM_001033614	RPS18	F: GATCCATTGGAGGGCAAGTC R: GCAGCAACTTTAATATACGCT	124
NM_001034034	GAPDH	F: TGACCCCTTCATTGACCTTC R: TACTCAGCACCAGCATCACC	130

* From Thering et al. [33]. All other sequences were designed using NCBI Primer BLAST.

### 2.13. Statistical Analysis

SAS (v 9.4) was used for statistical analysis. Changes in blood metabolites, mitochondrial measures, and oxidative stress and damage variables across lactation and in relation to milk yield were assessed using general linear mixed models with time (i.e., lactation stage) and milk yield as fixed effects and cow ID as a random effect. Additional general linear mixed models were run with milk composition fat, protein, and lactose individually as fixed effects. For gene expression, Ct values of reference genes were used as covariates in general linear mixed models to compare changes in gene expression between early to peak and early to late lactation. Data were checked for normality visually and through Komolgorov-Smirnov tests. Statistical significance was declared at *p* < 0.05. *p*-values between 0.05 and 0.1 were considered a tendency. Data are presented as either means or LSMeans ± SEM.

## 3. Results

### 3.1. Body Weight and Blood Metabolites

Body weight did not vary substantially across lactation (*p* = 0.94). Plasma glucose concentration increased across lactation whereas circulating NEFA concentration decreased across lactation. In contrast, BHB concentrations were similar across time (Table 2).

### 3.2. Mitochondrial Respiration

**Figure 1:**
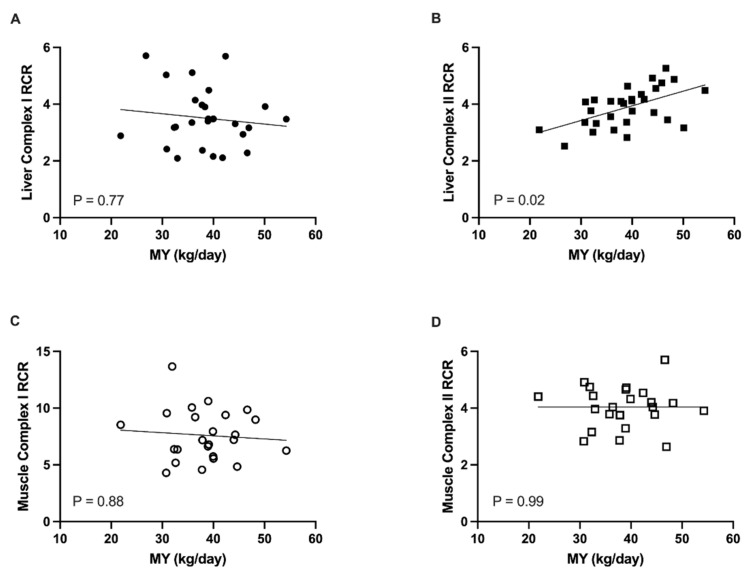
Mitochondrial respiratory control ratio (RCR) for (**A**) liver complex I, (**B**) liver complex II, (**C**) skeletal muscle complex I, and (**D**) skeletal muscle complex II in association with milk yield (MY). RCR, calculated as mitochondrial maximal to resting respiration rate, is a measure of mitochondrial coupling efficiency. Circles represent complex I mediated respiration and squares represent complex II respiration. Closed symbols indicate liver tissue whereas open symbols denote skeletal muscle tissue. MY was recorded as total kg produced the day before biopsy. Tissue biopsies were collected at 8 ± 2 DIM (early), 75 ± 4 DIM (peak), and 199 ± 6 DIM (late) lactation on 11 multiparous Holsteins per timepoint.

Despite a decrease in state 3 and 4 respiration using complex I substrates (pyruvate, malate, glutamate) from early to peak lactation (Supplementary Table S1), liver complex I RCR did not change across lactation (*p* = 0.26) and did not vary with MY (*p* = 0.77; Figure 1). Liver complex II RCR was positively associated with MY (*p* = 0.02; Figure 1) and decreased from peak to late lactation (peak, 4.20 ± 0.18; late, 3.68 ± 0.17; *p* = 0.049), but was similar between early and peak lactation (*p* = 0.11) and early and late lactation (*p* = 0.75). Muscle state 3 and 4 respiration using complex I substrates increased from early to peak lactation (Supplemental Table S1). However, muscle complex I RCR did not vary across time (*p* = 0.33) nor was there an association with MY (*p* = 0.88; Figure 1). Muscle state 3 and 4 respiration using succinate also increased from early to peak lactation (Supplemental Table S1). Muscle complex II RCR was similar across lactation (*p* = 0.54) and did not vary with MY (*p* = 0.99; Figure 1).

### 3.3. Oxidant Emission and Oxidative Stress

**Figure 2:**
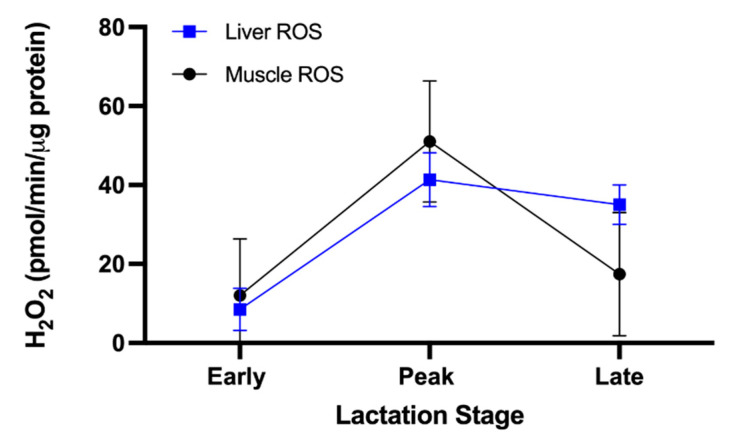
Oxidant emission in liver and skeletal muscle mitochondria across lactation. ROS were quantified from isolated mitochondria at early (8 ± 2 DIM), peak (75 ± 4 DIM), and late (199 ± 6 DIM) lactation from 11 multiparous Holsteins per timepoint. Data are presented as LSMeans ± SE. Liver ROS emissions increased significantly throughout lactation (*p* = 0.001).

Liver mitochondrial ROS increased across lactation from early to peak (*p* = 0.001; Figure 2) and early to late lactation (*p* = 0.001; Figure 2). No associations were found between liver ROS and MY (*p* = 0.28). There was a tendency for a negative association between muscle ROS and milk protein concentration (*p* = 0.09). Muscle ROS did not vary across lactation (*p* = 0.19) or with MY (*p* = 0.12; Figure 2).

**Figure 3:**
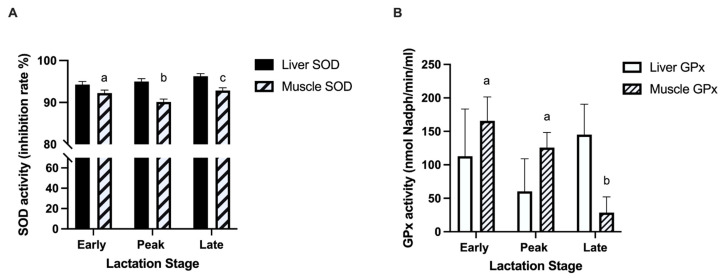
Antioxidant activity in liver and skeletal muscle across lactation. Antioxidant activity of (**A**) superoxide dismutase (SOD) and (**B**) glutathione peroxidase (GPx) were measured at early (8 ± 2 DIM), peak (75 ± 4 DIM), and late (199 ± 6 DIM) lactation from 11 multiparous Holsteins per timepoint. Disparate letters above bars represent significant differences between lactation stages for antioxidant activity in skeletal muscle tissue (*p* < 0.05).

Liver SOD activity tended to have a positive association with milk protein concentration (*p* = 0.05) and a negative association with milk fat (*p* = 0.10) but was not associated with MY (*p* = 0.44). Liver SOD activity was also similar across lactation (*p* = 0.13; Figure 3). Muscle SOD activity tended to have a negative association with MY (*p* = 0.07; Figure 3). Muscle SOD activity decreased from early to peak lactation (*p* = 0.05; Figure 3) before increasing to late lactation (*p* = 0.01; Figure 3). No relationships were observed between muscle SOD activity and milk composition (*p* > 0.10). Liver GPx activity did not change across lactation (*p* = 0.48; Figure 3) and was not associated with MY (*p* = 0.75). Muscle GPx activity had a significant positive association with milk fat composition (*p* = 0.01) and decreased across lactation (*p* < 0.01; Figure 3).

Liver MDA concentrations were very low across all samples and MDA was undetectable in muscle. Liver MDA was similar across lactation (*p* = 0.27) and was not associated with MY (*p* = 0.98) Liver protein carbonyl concentration also did not vary across lactation (*p* = 0.65) and was not associated with MY (*p* = 0.86). However, muscle protein carbonyl tended to have a positive association with milk fat composition (*p* = 0.07) and was lower at peak lactation than any other lactation stage (early, 0.82 ± 0.093 vs. peak 0.27 ± 0.088; *p* < 0.001; peak 0.27 ± 0.088 vs. late, 0.76 ± 0.085; *p* < 0.001). No associations were found between skeletal muscle protein carbonyl concentration and MY (*p* = 0.37).

### 3.4. Adenosine Nucleotides

The liver ADP/ATP ratio tended to decrease across lactation from early to late (early, 4.14 ± 0.80; late, 1.95 ± 0.75; *p* = 0.06) and from peak to late lactation (peak, 3.93 ± 0.67; late, 1.95 ± 0.75; *p* = 0.07). Liver ATP concentration tended to positively associate with milk yield (*p* = 0.10) and was significantly associated with milk lactose (*p* = 0.01). Muscle ADP/ATP ratios did not vary over time (*p* = 0.40) or with MY (*p* = 0.16) or milk components (all *p* > 0.25). Muscle ATP concentrations also did not differ between timepoints (*p* = 0.40) or with MY (*p* = 0.37) or milk composition (all *p* > 0.20). Muscle and liver AMP/ATP ratios were similar across time and were not associated with MY or components.

### 3.5. Gene Expression

**Figure 4:**
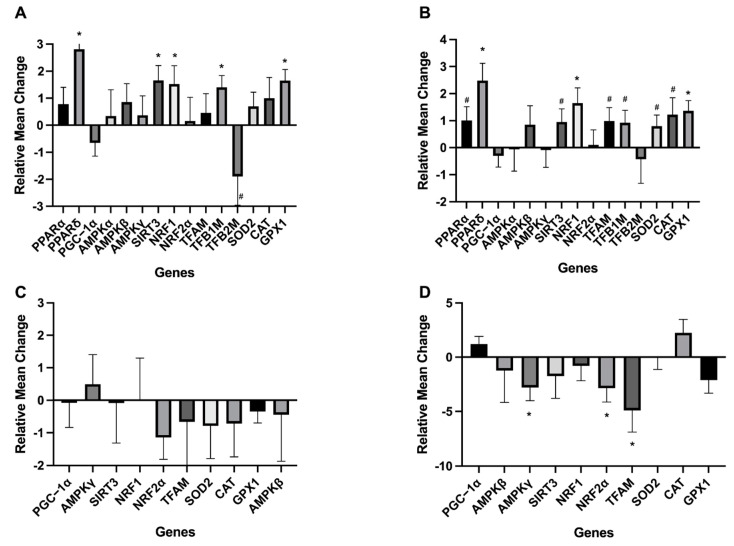
Changes in liver and skeletal muscle gene expression across lactation. Mean changes represent expression levels at peak or late lactation relative to early lactation: (**A**) gene expression in liver at peak lactation, (**B**) gene expression in liver at late lactation, (**C**) gene expression in skeletal muscle at peak lactation, (**D**) gene expression in skeletal muscle at late lactation. * denotes *p* < 0.05 and ^#^ denotes *p* < 0.10 and > 0.05. Expression of *PPARα*, *PPARδ*, *AMPKα*, *TFB1M*, and *TFB2M* were undetectable in skeletal muscle and thus are not presented here.

qRT-PCR was conducted to assess changes in the expression of genes involved in mito-nuclear communication and mitochondrial regulation (Figure 4). There was a tendency for liver *PPARα* to be upregulated from early to late lactation (*p* = 0.07). Liver *PPARδ*, *NRF1*, *TFB1M*, *SIRT3*, and *GPx* expression were upregulated as lactation progressed. *TFB2M* expression in the liver tended to decrease from early to peak lactation (*p* = 0.09) whereas liver *SOD* and *CAT* expression tended to increase from early to late lactation.

Expression of *PPARα*, *PPARδ*, *TFB1M*, *TFB2M* was undetectable in skeletal muscle. Muscle *NRF2* expression was downregulated from early to late lactation (*p* = 0.05), whereas *TFAM* expression was downregulated at late lactation relative to early (*p* = 0.04). *AMPKγ* expression decreased from early to late lactation in skeletal muscle (*p* = 0.04). The expression of all other analyzed genes was similar across lactation.

## 4. Discussion

Milk synthesis is a highly energetically demanding process for female mammals requiring coordinated metabolic adaptations across tissues [1]. Mammary mitochondria are integral to milk synthesis, with roles in the biosynthesis of milk precursors and energy production to fuel mammary maintenance, growth, and milk production [11]. However, functional adaptations in the mitochondria of extramammary tissues and the regulatory pathways involved have not yet been fully elucidated in the dairy cow. We hypothesized that mitochondria in the liver and skeletal muscle would undergo functional changes in respiratory capacity and oxidative status across lactation that may allow for coordinated metabolic changes to support shifting energy demand associated with milk synthesis. We assessed mitochondrial function at early, peak and late lactation in multiparous Holsteins and found that functional changes were tissue dependent. Liver showed changes in mitochondrial coupling and oxidant emission across lactation while skeletal muscle had increased incidence of oxidative damage with little change in mitochondrial function.

### 4.1. Mitochondrial Respiration

RCR was used as a general measure of mitochondrial coupling efficiency and was calculated as the ratio of the maximal mitochondrial respiration rate to the resting rate [34]. We found that liver complex I RCR was similar across lactation and was not associated with changes in milk yield. Similarly, Mowry et al. (2017) showed that mice at peak lactation had no change in liver or skeletal muscle RCRs relative to non-lactating animals. However, Hyatt et al. [18,19] found that liver complex I RCR in peak lactating rats was higher than nonreproductive females, but lower compared to pregnant females. Differences in lactation stage of the females and in glucose metabolism between ruminants and non-ruminants may explain, at least in part, discrepancies between our findings in cattle and those in rodents. Complex I efficiency is associated with glucose metabolism because the complex I cofactor, NADH, is produced as a byproduct of complete oxidation of glucose [35]. While monogastrics are typically not glucose limited because they readily absorb glucose across the GI tract, lactating ruminants absorb very little exogenous glucose and much of the available glucose is spared for milk lactose synthesis [36]. Consequently, we found that circulating glucose was substantially lower during early lactation as milk production was ramping up.

In our study, liver complex II RCR was positively associated with MY. Within the TCA cycle, succinate (considered a fat substrate) is converted to fumarate, producing FADH2 [35]. Thus, our results suggest that higher coupling of fat substrate oxidation to ATP production is positively correlated with milk production. NEFA was also highest during early lactation, associated with lipolysis from adipose tissue [4,37]. NEFA arriving at the liver can be used for fatty acid oxidation or converted to ketones [37]. Together, greater circulating NEFA early in lactation and the association between liver complex II RCR and MY suggest that as the milk yield and energy demand are increasing in early lactation, NEFA in the liver are prioritized as substrates for oxidative phosphorylation.

In contrast to liver RCR, skeletal muscle RCR did not vary across lactation or with changing MY. This may imply that the mitochondrial efficiency in this tissue is less affected by the shifting metabolic demand that is associated with lactation, corroborating studies in rodents [18,19,20]. The lack of observed changes in ETS coupling in skeletal muscle across lactation, may be associated with the muscle fiber type. In our study, samples were collected from the biceps femoris muscle. In humans, this muscle consists of similar proportions of type 1 and type 2 muscle fibers [38], which provides versatility in oxidative and glycolytic capacity within the muscle [39]. At the onset of lactation, a reduction in TCA cycle enzyme concentrations in skeletal muscle of lactating cattle is paired with the enrichment of glycolytic pathways [8]. Therefore, fiber type variation may allow for a switch from predominately oxidative to glycolytic metabolism, with little to no change in ETS coupling. Maximal respiration rates of skeletal muscle also decrease in response to diets that are high in fat [40]; thus, increasing adipose mobilization and circulating levels of NEFA during early lactation may prevent an increase in the oxidative capacity of muscle mitochondria. Furthermore, muscle fatty acid oxidation is reduced during the transition period in dairy cows, allowing fatty acid use by other tissues for energy and for milk synthesis [20].

### 4.2. Oxidative Stress

ROS production occurs when electrons leave the inner mitochondrial membrane during respiration, mostly from complexes I, II, and III of the ETS, and combine with molecular oxygen to form radicals [41]. This can be induced through ETS uncoupling proteins whereby electrons flow through the protein complexes but escape the ETS, resulting in dissipation of the proton motive force and limited ATP production [42]. Unregulated or excessive ROS formation can cause oxidative damage [42,43,44], such as protein carbonyl formation, lipid peroxidation, or damage to DNA [34,45,46].

In the present study, liver ROS emissions were lowest shortly after parturition and increased throughout lactation, contrasting earlier reports of greater circulating oxidant concentrations during the transition period in dairy cattle [47]. ROS concentrations increase with β-oxidation [48], which is elevated during the transition period, coinciding with lipolysis from adipose tissue and elevated peripheral NEFA concentrations [4,37]. Excess NEFA concentrations were linked to increased ROS and metabolic diseases, such as ketosis [21].

Oxidative stress occurs when there is an imbalance between ROS production and antioxidant expression or activity [49]. With the capacity to neutralize oxidants and prevent oxidative damage [24], antioxidant activity is expected to increase in response to elevated ROS production. In dairy cattle, antioxidant concentrations are highest during the transition period, especially immediately following parturition [47,50]. In our study, muscle SOD and GPx activity decreased across lactation and SOD was negatively associated with MY. SOD acts upon oxidants by converting radicals to hydrogen peroxides which are then broken down further by GPx or CAT [24]. Whereas antioxidant activity changed across lactation, the expression of *SOD*, *GPx*, and *CAT* in skeletal muscle did not, suggesting that improved antioxidant status is largely a product of enhanced activity rather than upregulation of antioxidant gene expression in this tissue. In skeletal muscle, the abundance of protein carbonyls, a marker of oxidative damage, was elevated at early and peak lactation even though antioxidant activity was higher during early lactation and muscle RCR and ROS emissions were similar across lactation. These lactation stages are characterized by increased protein turnover as proteolysis in skeletal muscle increases during the transition period and protein synthesis is upregulated towards dry off [8]. Thus, skeletal muscle remodeling at these stages might induce oxidative damage, despite no change in RCR.

Liver SOD and GPx activity were not highly variable across lactation, despite the increase in liver ROS levels. However, liver *SOD*, *CAT*, and *GPx* expression increased across lactation, likely stimulated by increasing ROS emissions. There was no evidence of liver oxidative damage as quantified by markers of lipid peroxidation and protein oxidation suggesting a sufficient balance between liver oxidants and antioxidants. In contrast to skeletal muscle, it appears that in liver tissue, the observed change in redox balance is associated with enhanced mRNA expression of antioxidants rather than marked changes in antioxidant activity. Alternatively, it is possible that other antioxidants, such as vitamin E, beta-carotene, as well as peroxiredoxins, and oxidative defense pathways that were not assessed in our study prevented excessive ROS accumulation and oxidative damage.

In our study, both liver and muscle antioxidant activity were associated with milk composition. Liver SOD activity was greater in cows producing milk with higher protein content and muscle GPx activity was positively associated with milk fat composition. There is a paucity of published research linking oxidative stress to milk composition, but one study found that mastitis induced by excess ROS production in the mammary gland negatively impacted milk protein concentrations [51]. It is possible that oxidative stress impairs the mammary gland’s ability to synthesize milk macronutrients.

### 4.3. Regulation of Mitochondrial Bioenergetics

ROS are important signaling molecules that are involved in cellular energy homeostasis [49]. ROS production, linked to a high AMP/ATP ratio, induces AMPK activation through phosphorylation [52]. High ADP/ATP ratios also signal low energy status and stimulate ATP production through AMPK activation [35]. In liver, the ADP/ATP ratio was highest in early lactation, and this was concomitant with elevated ROS emission whereas the AMP/ATP ratio did not vary with lactation stage. This suggests a higher proportion of ADP precursor being available for phosphorylation early as compared to later in lactation where ATP demands for milk production lessen.

AMPK has three subunits α, β, and γ that respond to changes in adenosine nucleotide concentrations [53]. We detected no changes in the mRNA expression of any of the AMPK subunits across lactation in the liver and a downregulation in one subunit across lactation in skeletal muscle. In the skeletal muscle, our findings support research that found that AMPK protein expression was highest directly after parturition, along with PGC-1α, to promote mitochondrial biogenesis in rodents with prolonged lactation [15]. However, due to various factors, such as divergent levels of regulatory control between genes and proteins and variation in protein stability, protein and gene expression may not necessarily be correlated [54]. Additionally, AMPK is activated by posttranslational modification and thus, enzymatic activity can be modulated without a change in gene expression.

Mitochondrial biogenesis is initiated when energy status is low by peroxisome proliferator-activated receptor gamma coactivator 1 (PGC-1), which activates nuclear PPARs [55]. In the present study, *PGC-1α* expression was consistent in the liver across lactation thus opposing the findings of Hadsell et al. [15], in which PGC-1α abundance within the mammary glands of mice was highest directly after parturition. In our study, liver *PPARδ* was upregulated for the duration of lactation. *PPARδ* is involved in the initiation of fatty acid oxidation [20,56], so a pattern of upregulation contradicts the decline in nutrient and energy demands that occurs late in lactation.

PGC-1α induces nuclear respiratory factor (NRF) expression, which upregulates mitochondrial transcription factors (mtTFs) [55,57] to increase the oxidative capacity and coupling of the ETS [22,23,58]. In our study, liver *NRF1* expression increased across lactation while expression of the mTFs, *TFAM* and *TFB1M*, were stable across lactation and *TFB2M* was slightly downregulated at peak lactation. Surprisingly, given the role of PGC-1α in the modulation of mTF transcription, we found that *PGC-1α* and *TFB2M* had contrasting patterns of expression across lactation, with *PGC-1α* increasing and *TFB2M* decreasing. However, TFB1M can modify RNA in addition to functioning as a transcription factor and thus may have a different expression pattern than TFB2M [59]. In skeletal muscle, the expression of *NRF2α* and *TFAM* decreased across lactation. In congruence with our muscle RCR data, *TFAM* was downregulated in late relative to early lactation, suggesting that mitochondrial oxidative capacity is not elevated across lactation, likely a strategy to conserve energy and nutrients for milk production.

OXPHOS capacity is also regulated through the activation of sirtuins (SIRT), which may activate TCA enzymes [60,61] to enhance ATP production. Herein, we observed an increase in liver *SIRT* expression across lactation along with an increase in liver coupling efficiency. In lactating dairy cattle, associations between SIRT expression and OXPHOS efficiency were documented in certain contexts, such as when cows were grazing versus fed a total mixed ration [62].

## 5. Conclusions

To our knowledge, this is the first study to assess mitochondrial respiratory capacity in extramammary tissues of the dairy cow across lactation. The mitochondria in the liver of lactating dairy cattle exhibited improved mitochondrial coupling efficiency of complex II-based substrates to increase ATP production as milk yield and energy demand rose. While oxidant emission increased across lactation, there was no evidence of liver oxidative damage, suggesting sufficient antioxidant activity to scavenge ROS. On the other hand, mitochondria in the skeletal muscle had minimal changes in respiratory capacity across lactation, yet oxidative damage was especially prevalent at early and late lactation. Our results suggest that liver mitochondrial function is enhanced during lactation, which is similar to prior studies of the mammary gland. However, the oxidative capacity of the skeletal muscle remained relatively constant across lactation, presumably to maintain muscle function without drawing upon the limited resources that are needed by the mammary gland for milk synthesis. Ultimately, understanding mitochondrial function and the associated pathways regulating mitochondrial proliferation and activity throughout lactation could lead to new strategies and therapies to mitigate the risk of metabolic diseases during transition and improve lactation performance.

## Figures and Tables

**Table 2:** Changes in blood metabolites across lactation.

Metabolites	Early Lactation	Peak Lactation	Late Lactation	*p*-Value (Early vs. Peak)	*p*-Value (Peak vs. Late)	*p*-Value (Early vs. Late)
Glucose (mg/dL)	61.38 ± 3.59	59.37 ± 3.40	85.75 ± 3.31	0.69	<0.0001	<0.0001
NEFA (mmol/L)	5.34 ± 2.15	4.61 ± 2.14	4.59 ± 2.14	<0.0001	0.89	<0.0001
BHB (μmol/L)	109.27 ± 18.00	69.47 ± 17.04	69.05 ± 16.57	0.1265	0.9866	0.11

Data are presented as LSMeans ± SEM. Blood samples were taken at 8 ± 2 d, 75 ± 4 d, 199 ± 6 d in milk on 11 cows per timepoint.

## Data Availability

Data are contained within the article and in the Supplementary materials.

## Supplementary Materials

The following are available online at https://www.mdpi.com/article/10.3390/ani11092647/s1, Table S1: Mitochondrial respiration in skeletal muscle and liver tissue across lactation.
