# Supplementary material for: Mitochondrial Bioenergetics of Extramammary Tissues in Lactating Dairy Cattle
Source: Animals (Basel). 2021 Sep 9;11(9):2647. doi: 10.3390/ani11092647 (PMC8467216; doi:10.3390/ani11092647)
Supplement: Supplementary file 1 [file animals-11-02647-s001.zip › animals-1314497-supplementary.pdf]

Mitochondrial bioenergetics of extramammary tissues in lactating dairy cattle

Victoria Favorit [1], Wendy Hood [2], Andreas Kavazis [3], Patricia Villamediana [1], Kang Nian Yap [2], Hailey A. Parry [3], and Amy Skibiel [1,*]

Department of Animal, Veterinary and Food Sciences, University of Idaho, Moscow, ID 83844, USA; favo4940@vandals.uidaho.edu (V.F.); pvillamediana@uidaho.edu (P.V.)
[2] Department of Biological Sciences, Auburn University, Auburn, AL 36849, USA; wrhood@auburn.edu (W.R.H.); kny0004@auburn.edu (K.N.Y.)
[3] School of Kinesiology, Auburn University, Auburn, AL 36849, USA; ank0012@auburn.edu (A.N.K.); hap0017@auburn.edu (H.A.P.)
* Correspondence: askibiel@uidaho.edu; Tel.: +208-885-1161

Supplementary Table S1. Mitochondrial respiration in skeletal muscle and liver tissue across lactation.

**Tissue**	**Respiration measure***	**Early Lactation**	**Peak Lactation**	**Late Lactation**	***P*-value (early vs. peak)**	***P*-value (peak vs. late)**	***P*-value (early vs. late)**
Liver	State 3 Complex I	33.63 ± 3.49	21.41 ± 3.66	32.84 ± 3.32	0.03	0.03	0.87
Liver	State 4 Complex I	10.86 ± 0.85	6.57 ± 0.89	8.53 ± 0.80	0.002	0.13	0.05
Liver	State 3 Complex II	63.28 ± 20.67	48.08 ± 18.42	96.37 ± 17.95	0.59	0.08	0.23
Liver	State 4 Complex II	17.08 ± 6.74	10.84 ± 6.01	28.84 ± 5.86	0.50	0.05	0.19
Muscle	State 3 Complex I	34.42 ± 9.97	89.02 ± 10.35	39.87 ± 10.13	0.001	0.004	0.70
Muscle	State 4 Complex I	4.54 ± 1.53	13.41 ± 1.59	5.48 ± 1.56	<0.001	0.003	0.67
Muscle	State 3 Complex II	36.70 ± 23.42	127.78 ± 21.67	68.76 ± 23.97	0.01	0.10	0.34
Muscle	State 4 Complex II	9.94 ± 7.95	33.76 ± 7.36	17.72 ± 8.14	0.04	0.18	0.50

* Mitochondrial respiration in pmolO_2/min/μg mitochondrial protein. Pyruvate, malate, and glutamate were used as complex I substrates. Succinate was used as the complex II substrate. Tissue biopsies were collected at 8 ± 2 DIM (early), 75 ± 4 DIM (peak), and 199 ± 6 DIM (late) lactation on 11 multiparous Holsteins per timepoint. Data are presented as LSMeans ± SEM.
